# Real-Time Sound Source Localization for Low-Power IoT Devices Based on Multi-Stream CNN

**DOI:** 10.3390/s22124650

**Published:** 2022-06-20

**Authors:** Jungbeom Ko, Hyunchul Kim, Jungsuk Kim

**Affiliations:** 1 Department of Health Sciences & Technology, Gachon Advanced Institute for Health Sciences & Technology (GAIHST), Gachon University, Incheon 21936, Korea; rhwndqja@gachon.ac.kr; 2 School of Information, University of California, 102 South Hall 4600, Berkeley, CA 94720, USA; 3 Department of Biomedical Engineering, Gachon University, 191 Hambakmoe-ro, Incheon 21936, Korea

**Keywords:** sound source localization, deep learning, multi-stream CNN, IoT device

## Abstract

Voice-activated artificial intelligence (AI) technology has advanced rapidly and is being adopted in various devices such as smart speakers and display products, which enable users to multitask without touching the devices. However, most devices equipped with cameras and displays lack mobility; therefore, users cannot avoid touching them for face-to-face interactions, which contradicts the voice-activated AI philosophy. In this paper, we propose a deep neural network-based real-time sound source localization (SSL) model for low-power internet of things (IoT) devices based on microphone arrays and present a prototype implemented on actual IoT devices. The proposed SSL model delivers multi-channel acoustic data to parallel convolutional neural network layers in the form of multiple streams to capture the unique delay patterns for the low-, mid-, and high-frequency ranges, and estimates the fine and coarse location of voices. The model adapted in this study achieved an accuracy of 91.41% on fine location estimation and a direction of arrival error of 7.43° on noisy data. It achieved a processing time of 7.811 ms per 40 ms samples on the Raspberry Pi 4B. The proposed model can be applied to a camera-based humanoid robot that mimics the manner in which humans react to trigger voices in crowded environments.

## 1. Introduction

**Figure 1:**
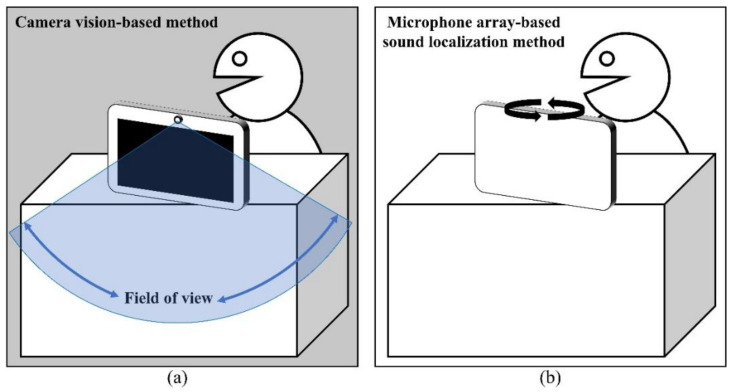
(**a**) Limitation of the camera vision-based method is that it cannot track if the user deviates from the field of view of the camera; (**b**) The advantage of the microphone array-based sound localization method is that it can track the user by estimating the direction through sound regardless of any direction.

Voice recognition-based systems, such as smart speakers and displays, recognize the voices of registered users and perform tasks by extracting semantic information from their speech. These systems allow users to search for information or run applications in situations where they cannot use their hands to control the devices. Because of this convenience, they have been widely adopted in recent smart devices as a core function, and customers actively use these smart devices while using home surveillance cameras, watching videos, and making video calls through apps installed on their devices. However, because of the nature of static devices with limited viewing angle cameras and displays, users are constrained to stay within a certain range while interacting with these devices for video calls or watching videos; otherwise, the direction of the device must be adjusted by hand, which clearly violates the purpose of these devices and reduces user satisfaction (Figure 1).

The abovementioned mobility limitation can be resolved by tracking the user’s location in relation to the device. This can be achieved by detecting the user’s location using a camera or multi-channel microphone arrays, which mimic human recognition systems. The camera-based method appears to be a more intuitive and convincing user location detection method. In particular, owing to the recent developments in convolutional neural networks (CNNs) [1,2,3], object detection accuracy has improved significantly, and user location detection is no longer a challenge. However, this method has a fundamental limitation in that it cannot provide a complete solution to the user location detection problem. First, users do not feel comfortable owing to the feeling of being observed, which is a potential privacy issue. Second, if users are located in a different room or space that is physically separated by a wall, the device fails to provide any service. Imagine a situation wherein a user accidentally falls down in a room next to where the device is located. If the device relies only on a camera, it cannot determine the location of the accident. Finally, running CNN-based object detection algorithms on IoT devices is costly even without an expensive GPU; this can be burdensome to the user.

The microphone array-based sound localization method can be implemented using an analytic sound source localization (SSL) algorithm such as in [4,5,6,7,8,9]. Unlike the camera vision-based method, this method has the advantage of users not worrying about privacy issues or space limitations. The traditional sound source localization method locates the sound source by processing the phase and amplitude differences of the sound signal received at each microphone array. However, the performance of the traditional solution decays in an environment with noise and reverberation [4,5] owing to the difficulty of modeling noise and interference, which depends on the geometry of the user space. Recently, several studies have been conducted using deep neural networks (DNNs) [6,7,8,9]. In [6], estimating the sound source location was treated as a classification problem. The location of the sound source was estimated by dividing 360° into 360 classes. A spectrogram of multi-channel acoustic data was used as the input, and a DNN modified from ResNet [10], which showed excellent performance in image classification, was used. In [7], researchers introduced a method for finding the azimuth and elevation of a sound source using a stacked convolutional and recurrent neural network. The entire network was divided into two: the first network receives a spectrogram of multi-channel acoustic data as an input and generates a spatial pseudo-spectrum (SPS) as an intermediate output, and the second receives it as an input to estimate the azimuth and elevation of the sound source. Unlike previous studies, [8,9] used an end-to-end technique to extract features directly from multi-channel raw acoustic data. In [8], the 3D coordinates of the sound source were predicted using a simple CNN model, and in [9], the distance and azimuth of the sound source were predicted using the DNN proposed for audio classification using the raw acoustic data in [11]. These studies demonstrated that the SSL based on DNNs significantly improved the accuracy and robustness of the model compared with traditional signal processing-based methods. Furthermore, [12] used a CNN to analyze the speech intelligibility, which shows the suitability of a CNN for processing acoustic data.

However, the DNN-based approaches mentioned thus far are unsuitable for practical IoT systems that typically lack sufficient computation power. To run the DNN-based SSL on IoT devices in real time, the number of hyperparameters in the DNN must be limited while satisfying the minimum performance requirement. Therefore, in this study, a novel CNN-based real-time SSL model was considered for real-time operation in low-power IoT devices. The proposed CNN model has multi-stream (MS) blocks that comprises convolution layers of various sizes connected in parallel that can capture the low-, medium-, and high-frequency component features from multi-channel acoustic data. Owing to the parallel structure of the MS block, the model can reduce the number of parameters and computations without sacrificing performance. For the model training and testing datasets, the TIMIT database [13] was simulated with a room impulse response (RIR) generator to construct multi-channel acoustic datasets in various directions, heights, and distances.

## 2. Materials and Methods

### 2.1. Overall System Architecture

**Figure 2:**
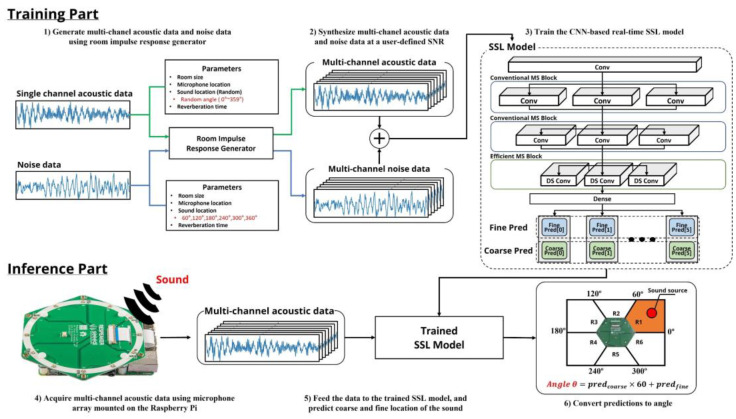
System overview of real-time SSL system using IoT devices.

The proposed system can be divided into two parts, as illustrated in Figure 2. In the training part, actual single-channel sound data are collected as the main sound source, and multi-channel acoustic data reflecting various room conditions are generated using the image method [14] based on the RIR generator. This generator tool can provide control over different room sizes, microphone locations, sound source locations, and reverberation times to define various room environment impulse response models, and pseudo multi-channel acoustic data can be created by convolving the RIR impulse response with the sound source data. Subsequently, the pseudo multi-channel data are synthesized with noise data containing a buzzing sound in a cafe, with a certain probability during the model training process at a user-defined signal-to-noise (SNR) ratio for data augmentation, thereby improving the model performance in practical situations. Finally, these data were used to train the proposed CNN-based real-time SSL model, which does not require the data to be transformed into handcrafted feature vectors, such as spectrograms or mel-frequency cepstral coefficients (MFCCs), which have been widely used in acoustic data model training. This allows the model to capture more unbiased sound characteristics for localization directly from raw data [15].

The inference part was implemented on an IoT device for real-time operation, and the angle of the sound wave arrival was predicted by processing multi-channel acoustic data with a microphone array module on the IoT system. The IoT device and microphone array module used in this system are Raspberry Pi 4B (Raspberry foundation, Cambridge, UK) and Respeaker 6-microphone circular array (Seeed Technology Co., Ltd., Shenzhen, China), respectively. They were selected because of their advantages of reasonable price and low power consumption, which enables portable device applications. Designing a compact DNN model for real-time operation is vital because a Raspberry Pi 4B operates at considerably slower clock speeds than a desktop PC and does not have a powerful GPU. The Respeaker 6-microphone circular array is a module compatible with the Raspberry PI; it comprises six microphones arranged in a circle. The multi-channel acoustic data from the quantized microphone array are fed to the proposed CNN model, and the model predicts the continuous sound location in two coarse and fine prediction processes.

### 2.2. Dataset

The multi-channel acoustic dataset for SSL model training was constructed by synthesizing simulated room impulses created using the image method-based [14] RIR generator and TIMIT database [13]. TIMIT is a dataset comprising broadband recordings, in which 630 speakers with 8 dialects read 10 sentences. Similar to [9], clean speech corresponding to dialect 8 (DR8) was used for synthesis. Image method-based RIR generators are widely used in the SSL field because they can easily generate multi-channel acoustic data profiles at various distances, heights, and angles by applying simulated room impulses to the original single-channel acoustic data. The RIR generator requires sampling rate, receiver positions, source position, room dimensions, and reverberation time.

**Figure 3:**
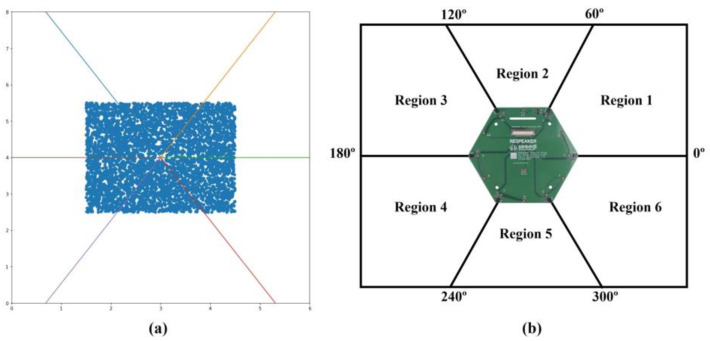
(**a**) A 2D schematic map of the microphone array and sound sources. The blue dots indicate sound sources, and the colored lines are extension lines between the center of the microphone array and each microphone; (**b**) Regions were divided according to the locations of the microphones.

The sampling rate in this study was 16 kHz, and the receiver positions matched the positions of the microphones on the Respeaker 6-microphone circular array module. The source positions of each sample were randomly assigned an angle from 0 to 360° and distance from 0.1 to 2.12 m, as shown in Figure 3a. The room dimensions were 6×8×3 for the convenience of the study, considering a typical room size. The reverberant time was set to 400 ms, considering that the average reverberation time of a residential space with furniture ranges from 300 to 500 ms. The room impulse created according to these settings was convolved with clean speech data to finally transform into multi-channel acoustic data. The generated data were split according to the input size of the SSL model. Table 1 presents the overall dataset configuration. Most studies used an input duration of at least 500 ms, whereas we used a shorter input of 40 ms such that the SSL model could respond more quickly to changes in the direction of the sound source. The input has a short duration; therefore, a threshold was applied to the average amplitude of the data as an absolute value to handle silent data in which almost no sound is heard; these data were then filtered. As a final training step, the data were augmented by synthesizing a sound source with noise data located at 60°, 120°, 180°, 240°, 300°, and 360° with the desired SNR at a random probability. This strategy was used to increase data diversity, prevent overfitting, and improve model performance to obtain robust models in the presence of noise.

### 2.3. Classification and Regression together

Among the several methods that estimate the location of a sound source, the most common approach based on DNNs is to treat the localization problem as a classification problem by discretizing angles into classes. Several studies [6,7] have shown that classification approaches based on DNNs provide more robust and accurate results compared with traditional signal-processing-based SSL algorithms [4,5].

However, this type of approach has a fundamental limitation in that it requires many classes (up to 360) to estimate the angle of arrival (AoA) at a fine resolution, such as 1°. In the case of a compact DNN model, this causes overfitting because the number of parameters in the dense layer is more than 50% of that in the entire model. Thus, the classification-based approach cannot be used directly in our application. Therefore, we adopted a hybrid method that utilizes classification and regression together, similar to [9]. In the proposed method, the final location of the sound source is generated by estimating the region where the sound source exists through classification, and the fine angle through regression.

As shown in Figure 3b, the 360° space around the microphone array was divided into 6 regions. Therefore, the total output of our model is a 12-dimensional vector (classification and regression output for each region), which requires significantly fewer parameters and computations compared with classification-based methods. Finally, using this method, the angle resolution can be maintained using a small number of parameters.

### 2.4. Conventional Multi-Stream (MS) Block

**Figure 4:**
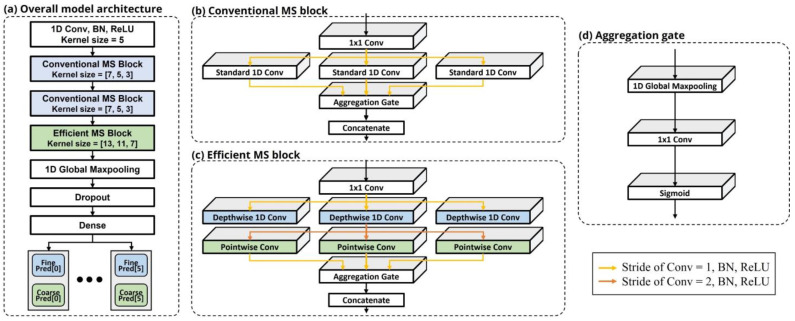
(**a**) Block diagram of the model architecture, (**b**) conventional MS block, (**c**) efficient MS block, and (**d**) aggregation gate. The colored arrows indicate the stride of convolution, the subsequent batch normalization (BN) [17], and ReLU [18].

The conventional MS block is designed to capture the features of various scales from multi-channel acoustic data. The signal for each channel of multi-channel acoustic data can be defined by the following equation

(1) $$y_{i}=w_{i}(t)*x(t-\tau_{i})+n_{i}(t),\{\begin{array}{l} n_{i}:\text{received}\;\text{signal}\;\text{at}\;i\mathrm{th}\;\text{channel}\\ w_{i}(t):i\mathrm{th}\;\text{channel}\;\text{impulse}\;\text{response}\\ x(t):\;\text{sound}\;\text{source}\;\text{signal}\\ \tau_{i}:\;\text{time}\;\text{delay}\;\text{of}\;i\mathrm{th}\;\text{of}\;\text{channel}\\ n_{i}(t):\;\text{noise}\;\text{at}\;i\mathrm{th}\;\text{channel} \end{array}$$

where w is not necessarily linear and can be a time varying parameter. In addition, channel response w and signal x are unknown in the sound localization problem. It is extremely difficult to estimate channel impulse response and channel dependent time delay. The feature of multi-channel acoustic data is a delay pattern that appears on the waveform owing to not only the difference in time for sound to arrive at each microphone in the microphone array, but also external factors such as noise. Because the acoustic signal is the sum of periodic functions with various frequencies, these delay patterns occur on multiple scales. In addition, as signals are absorbed and reflected by objects such as furniture and people, the delay patterns become more complex. To capture them, a conventional MS block was constructed with a series of standard 1D convolutions of different sizes in parallel. Figure 4b illustrates the structure of a conventional MS block. The input dimension of the conventional MS block is reduced by a 1×1 convolution and then passed to each CNN stream, which processes the low-, mid-, and high-frequency components to capture the unique delay pattern. The use of convolution layers of various sizes was introduced in [16], which is known to handle multiple scale features.

Below the series of standard 1D convolutions is an aggregation gate (AG), which is a neural network for combining features extracted from each CNN stream. It was proposed in [19] and was designed to fuse multi-scale feature maps by channel-wise weights. The research in [19] was for person reidentification using image data; therefore, we transformed the architecture of the AG to fit the acoustic data domain. Figure 4d illustrates the architecture of the AG.

### 2.5. Efficient Multi-Stream (MS) Block

**Figure 5:**
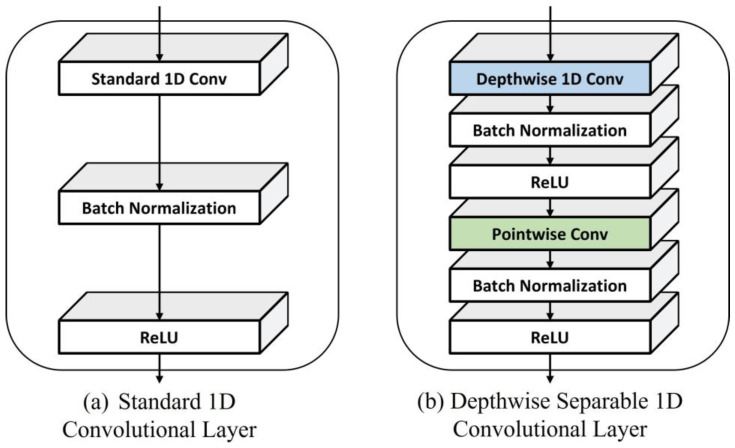
Comparison between (**a**) standard 1D Convolution and (**b**) depth-wise separable 1D convolution.

An efficient MS block is a unit in which the standard 1D convolutions of each stream of the conventional MS block are replaced with factorized 1D convolutions to reduce the computation and number of parameters. Figure 4c illustrates the architecture of an efficient MS block. We adopted depth-wise separable convolution [20] as a convolution factorizing method (Figure 5). Depth-wise separable convolution is known to significantly reduce the computation with a small performance degradation. The input of our SSL model is data in the time domain; therefore, we used depth-wise separable 1D convolution instead of the standard depth-wise separable 2D convolution. The stride of the depth-wise 1D convolution was set to 2 to further reduce the computation. Let the input and output channels be $C_{in}$ and $C_{out}$, respectively, the input and output widths be $W_{in}$ and $W_{out}$, respectively, and the filter size of the convolution be k. The computation of the standard 1D convolution is $C_{out}\times C_{in}\times W_{out}\times k$, and that of the depth-wise separable 1D convolution is $C_{out}\times W_{out}\times \left(C_{in}\times k\right)$.

Despite the computation reduction effect of the efficient MS block, we decided to use it only in the last layer. This is because of the performance degradation problem that occurs when an efficient MS block is used in an early layer. The size of the convolution filters of the efficient MS block was set greater than that of the conventional MS block. This shows the result of compensating for performance degradation with only a small increase in computation. Performance degradation when using factorized convolution was reported in [21]. They proposed a method to factorize an n×n convolution filter into 1×n and n×1 filters. They reported that the performance of the model was good when a larger filter was used and performance degradation was seen when the factorized convolution was used in the early layer.

### 2.6. Model Architecture

The SSL model can be roughly divided into the front CNN layer, conventional MS block, efficient MS block, AG, global max-pooling, dropout, and dense layers, as shown in Figure 4a. First, the front CNN layer extracts basic features and reduces redundant data using convolution filters with 32 kernels of size 5 and stride of 2. The output of the front CNN layer is fed to the conventional MS block, which reduces the input dimension using 1×1 convolution and extracts multiple scales of features by three 1D standard convolutions with different filter sizes (7, 5, and 3) and a stride of 2. The multiple scales of the features are then concatenated and combined after passing the shared AG. The use of an AG enables the fusion of features on multiple scales. Below the two conventional MS blocks is an efficient MS block, in which the 1D standard convolutions are replaced with depth-wise separable convolutions with larger filter sizes (13, 11, and 7). The reason for this design is that it places only one efficient MS block, which has larger filter sizes, at the end to minimize the performance degradation, as mentioned in Section 2.5. The output of the efficient MS block is passed to the global max-pooling and transformed into a 144-dimensional vector. To reduce overfitting and obtain a well-generalized model, dropout [22] is applied, which is a regularization method that ignores a certain percentage of units during training. After the 144-dimensional vector passes through the dropout, two types of final outputs are generated in the dense layer. The first is coarse location, which determines which microphone region the sound originates from, and the other is a fine estimation of the angle the sound locates in each microphone region. Table 2 lists the shapes of the inputs and outputs in each block.

### 2.7. Performance Evaluation

The performance indicators used in this study were the direction of arrival (DOA) error, accuracy (ACC), and inference time per sample. First, the DOA error is a metric representing the average of the difference between the actual and predicted angles and is calculated using the following equation

(2) $$\mathrm{DOA}\;\mathrm{error}=\frac{1}{N}\overset{N}{\underset{i=1}{\sum }}\left|\theta_{i}-\hat{\theta_{i}}\right|,$$

where θ and $\hat{\theta }$ are the ground truth and predicted angles, respectively. N is the total number of samples.

Furthermore, ACC is the most common metric used to evaluate the performance of a classification model. Our SSL model classifies the region to which the sound source belongs; therefore, ACC can be used as a metric. However, even if the predicted region is wrong, it may be closer in angle; therefore, it is unsuitable for direct use. Thus, the ACC in this study was calculated by thresholding the difference between θ and $\hat{\theta }$ to differentiate between true and false cases. Threshold T in this study was 10, similar to that in [10].

(3) $$\mathrm{ACC}=\frac{1}{N}\overset{N}{\underset{i=1}{\sum }}f\left(\theta_{i},\;\hat{\theta_{i}}\right),\;f\left(x,\;y\right)=\{\begin{array}{c} 1,\;\left|x-y\right|\leq T\\ 0,\;\left|x-y\right|>T \end{array}$$

The inference time per sample indicates the time required for the model to process one sample. This is used to verify the effect of reducing the inference time caused by using the efficient MS block, and whether the real-time operation of the model in IoT devices is possible.

## 3. Results

The synthetic multi-channel acoustic dataset was divided by a ratio of 8:1:1 for training, validation, and testing, respectively. We used the hyperparameter settings listed in Table 3. The SSL model was trained using the Adam optimizer. The initial learning rate was 0.01, and if the training loss was maintained for a certain period, the learning rate was divided by 10. The total number of training epochs was 30 with a batch size of 256. The loss during training was calculated using the following equations:

(4) $$L_{coarse}\left(y,\;\hat{y}\right)=-\overset{C}{\underset{i=1}{\sum }}y_{i}\;\log\left(\frac{\exp\left(\hat{y_{i}}\right)}{\sum_{j=1}^{C}\exp\hat{y_{j}}}\right)$$

(5) $$L_{fine}\left(y,\;\hat{y}\right)={\left(y_{i}-\log\left(\frac{\exp{\hat{y}}_{i}}{\exp{\hat{y}}_{i}+1}\right)\right)}^{2},\;i=\mathrm{argmax}\left(y\right)$$

(6) $$L=\alpha \times L_{coarse}\left(y_{\mathrm{coarse}},\;{\hat{y}}_{coarse}\right)+\beta \times L_{fine}\left(y_{fine},\;{\hat{y}}_{fine}\right)$$

Here, $y_{coarse}$ and ${\hat{y}}_{coarse}$ are the ground truth and prediction of the region classification, respectively, and $y_{fine}$ and ${\hat{y}}_{fine}$ are the ground truth and prediction of the fine angle regression, respectively. C is the number of the regions divided according to the position of the microphone. $L_{coarse}$ is the softmax cross-entropy (CE) loss for region classification, and $L_{fine}$ is the sigmoid mean squared error (MSE) loss for fine angle regression. α and β are loss scaling factors such that classification and regression performances can be learned in a balanced manner.

The performance of the model was measured by comparing each metric in the test data with the SNR combination of the training data. After determining the best SNR combination for the training data, we compared the performance of the trained models composed of different types and sizes of convolution filters. For the inference time measurement, we ran inference using Pytorch [23] and TVM [24] on a desktop CPU (Intel Core i7-7700K) and Raspberry Pi 4B.

### 3.1. Model Performance Measurements

First, we compared the performance of the models trained with the data of different SNR combinations. Table 4 presents the ACCs and DOA errors in the test data with SNRs of 30, 20, 10, and 0 dB, according to the SNRs of the training data. As seen from the performance difference between rows 1 and 5, and rows 2, 3, 4, and 6, the absence of 0 dB training data results in significant performance degradation. Because the proposed system should be robust to external noise for useability in a real environment, we chose the training SNR combination of row 5, which achieved the highest ACC in the test data of 0 dB SNR as the base in the following experiments.

**Figure 6:**
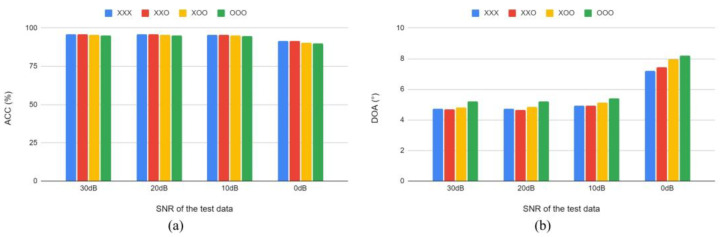
Comparison between (**a**) ACC and (**b**) DOA error in test data according to the type of block composing the SSL model. Here, “O” indicates efficient MS blocks, and “X” indicates conventional MS blocks.

After fixing the training SNR combination, we studied the influence of the block type in the model. As illustrated in Figure 6, the use of efficient MS blocks resulted in performance degradation. A comparison of the “XXX” model using only conventional MS blocks with the other rows in Table 4 reveals that the results are worse when more efficient MS blocks are used. However, the “XXO” model, which uses an efficient MS block only in the last layer, achieves better results in the test data with SNRs of 10, 20, and 30 dB. The “XXO” model represents the proposed basic model.

Table 5 lists the effect of the convolution filter size on the efficiency of the MS block. By expanding the size of the filter from (3, 5, 7) to (7, 9, 13), we achieved a significant performance improvement for all test data. In particular, the ACC increased by 0.7% in the test data of 0 dB SNR. Nevertheless, the increase in computation owing to the filter size expansion was only 0.008% of the total computation because depth-wise separable convolutions were used.

Additionally, we tested our basic model using a real-world dataset. This dataset was collected by recording the sounds of reading sentences using the Respeaker 6-microphone circular array. The distance to the microphone array was 0.5 m, and the room dimensions were 4×6×3. The total number of samples of the dataset is 1188. The model showed an ACC of 86.11% and DOA error of 6.14°. Considering that the speaker of the real-world dataset was not in the training data and the dimension of the room was different, this result shows that the model had a good generalization level.

### 3.2. Model Inference Time Measurements

**Figure 7:**
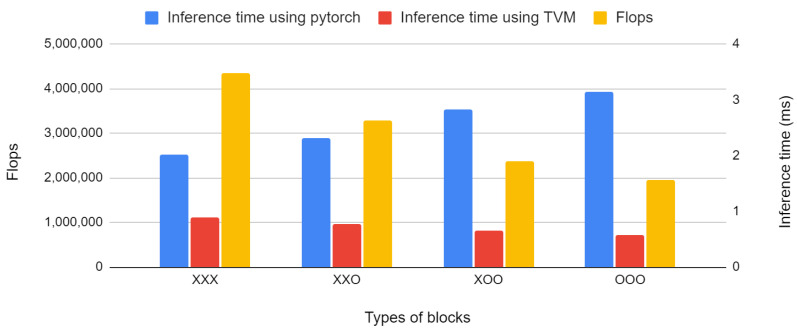
Comparison between the inference times using a desktop CPU according to the type of block composing the SSL model and the framework used for inference.

Both Pytorch and TVM used for inference time measurements are state-of-the-art deep learning frameworks, but they showed a large difference in inference time when depth-wise separable convolutions were used. Theoretically, replacing conventional MS blocks with efficient MS blocks reduces the amount of computation and inference time. However, as shown in Figure 7, in the case of inference with Pytorch, the computation reduced, but the inference time was longer. This occurs when depth-wise convolution is used in Pytorch, as reported in [25]. However, in the case of inference using TVM, the inference time was reduced. Based on the model in column 1, in which the efficient MS block was not used, the amounts of computation and inference time of our basic model (column 2) were reduced by 25% and 14%, respectively.

Table 6 shows the inference time per sample according to the batch size using Pytorch and TVM on the Raspberry Pi 4B. When running inference using Pytorch, and when the batch size was larger than 16, the inference time was 7.677 ms. However, when the batch size was smaller than 16, a considerably longer inference time was observed (approximately 56 times longer when the batch size was 1 than when the batch size was 16). In contrast, for inference using TVM, we had inference times of 7.811 and 5.772 ms when the batch sizes were 1 and 16, respectively. This result proves that our SSL model can be run satisfactorily in real-time on IoT devices.

## 4. Conclusions

In this study, we proposed a robust and efficient SSL model implemented on an IoT device for real-time operation. First, the basic model was constructed based on a multi-stream CNN architecture to capture the unique waveform patterns from the low, mid, and high frequencies of the acoustic data, which indicate the sound source orientation. This model was then augmented by applying synthesized data with the interference noise component, which improved the detection performance in noisy environments. Then, the basic multi-stream CNN architecture was optimized by replacing the conventional convolution layer with an efficient MS block that utilizes depth-wise separable convolutions to reduce the number of hyperparameters and computation cost. However, replacing the entire convolution layer with an efficient MS block caused an ACC degradation of approximately 2% in the 0 dB SNR acoustic data. To resolve this problem, we expanded the size of the convolution filter and placed an efficient MS block only in the last layer by referring to the results in [21].

The evaluation result of the final model reveals that this model has an accuracy greater than 90% even with 0 dB SNR acoustic data, and the inference time evaluation on the Raspberry Pi 4B shows an inference time of 7.811 ms per 40 ms sample on synthesized data. This test result indicates that the proposed model is robust and can be used for real-time operation on IoT devices. In our future work, we plan to extend the model to detect multiple sound sources and the elevation angles of sound sources on small IoT devices with ACC higher than 90%.

## Figures and Tables

**Table 1:** Dataset configuration.

Item	Value
Number of speakers	220
Sampling rate	16 kHz
Room size	6×8×3 (m)
Reverberation time	0.4 s
Distance	0.1 m–2.12 m
Angle	0–360°
Input duration	40 ms
Total number of samples	90,767

**Table 2:** Shapes of inputs and outputs in each block of the SSL model. In the input and output column, the tuple of two number denotes the width and channels, respectively.

Type of Module	Input	Output
Conv block	(640, 8)	(320, 32)
Conventional MS block	(320, 32)	(160, 48)
Conventional MS block	(160, 48)	(80, 96)
Efficient MS block	(80, 96)	(40, 144)
Global max-pooling	(40, 144)	(1, 144)
Dense	(1, 144)	(1, 12)

**Table 3:** Training hyperparameter settings of the SSL model.

Hyperparameter	Value
Total number of epochs	30
Batch size	256
Optimizer	Adam
Learning rate (LR)	0.01

**Table 4:** Performance of angle estimation using basic SSL model in test data according to the SNRs of the training data.

Row Number	Training SNR (dB)	Test SNR							
		30 dB		20 dB		10 dB		0 dB	
		ACC (%)	DOA (°)	ACC (%)	DOA (°)	ACC (%)	DOA (°)	ACC (%)	DOA (°)
1	30, 20, 10, 0	96.01	4.6436	95.95	4.7519	95.63	4.9653	88	9.0297
2	30	96.53	3.9954	96.52	4.0413	92.32	6.5589	33.58	58.1142
3	20	96.67	3.8748	96.62	3.9359	93.19	5.96	33.61	59.6756
4	10	96.41	4.0014	96.39	4.0832	95.79	4.5159	55.28	36.6573
5	0	96.07	4.6869	96.09	4.6663	95.72	4.9086	91.41	7.4289
6	X	96.81	3.9654	96.68	4.0048	92.41	6.3149	32.99	57.9033

**Table 5:** Performance of angle estimation in test data according to the size of the convolution filter.

Types of Blocks (Kernel Sizes)	Test SNR								Computation
	30 dB		20 dB		10 dB		0 dB		
	ACC (%)	DOA (°)	ACC (%)	DOA (°)	ACC (%)	DOA (°)	ACC (%)	DOA (°)	
XXO (3, 5, 7)	95.91	4.7539	95.85	4.7132	95.52	4.9142	90.74	7.6871	3,258,868
XXO (7, 9, 13)	96.07	4.6869	96.09	4.6663	95.72	4.9086	91.41	7.4289	3,285,748

**Table 6:** Comparison of inference times using Raspberry Pi 4B according to the batch size and framework used for inference.

Item	Batch Size	
	1	16
Inference time (ms) using Pytorch	426.93	7.677
Inference time (ms) using TVM	7.811	5.772

## Data Availability

The data used in this study are included in this article.

## Abbreviations

The following abbreviations are used in this manuscript:

SSL	Sound Source Localization
IoT	Internet of Things
DNN	Deep Neural Network
DOA	Direction of Arrival
SPS	Spatial Pseudo-Spectrum
CNN	Convolutional Neural Network
MS	Multi-Stream
AG	Aggregation Gate
RIR	Room Impulse Response
SNR	Signal-to-Noise Ratio
MFCC	Mel-Frequency Cepstral Coefficient
AoA	Angle of Arrival
ACC	Accuracy
ReLU	Rectified Linear Unit
BN	Batch Normalization
